# Arteriolosclerosis and LATE‐NC: two common and inter‐related contributors to Alzheimer’s‐type dementia

**DOI:** 10.1002/alz.085805

**Published:** 2025-01-03

**Authors:** Peter T Nelson, Yuriko Katsumata

**Affiliations:** ^1^ College of Medicine, University of Kentucky, Lexington, KY USA; ^2^ College of Public Health, University of Kentucky, Lexington, KY USA; ^3^ Sanders‐Brown Center on Aging, University of Kentucky, Lexington, KY USA; ^4^ University of Kentucky, Lexington, KY USA

## Abstract

**Background:**

Brain arteriolosclerosis (B‐ASC) is a pathologic hallmark characterized by dysmorphic brain arteriolar wall thickening. B‐ASC is a common finding at autopsy in aged persons – some degree of B‐ASC is seen in >80% of brains beyond age 80 years – and is associated with cognitive impairment. Hypertension and diabetes are widely recognized as risk factors for B‐ASC. We and others have also found that B‐ASC is associated with limbic‐predominant age‐related TDP‐43 encephalopathy neuropathologic change (LATE‐NC) and hippocampal sclerosis of aging (HS‐A). Here we re‐test this association using updated data from the National Alzheimer’s Coordinating Center (NACC) Neuropathology Data set.

**Method:**

Participants were assessed using the standardized NACC Uniform Data Set (UDS) at their local ADRC approximately annually, including participant demographics. Participants over 60 years of age at death (last seen during or after 2010) who met the study’s eligibility criteria were selected from the June 2023 (NACC62) data freeze. TDP‐43 neuropathologies were specified in three brain regions: amygdala, entorhinal/inferior temporal cortex and/or hippocampus, and neocortex. For cerebrovascular pathologies, data were available on arteriolosclerosis (none, mild, moderate, and severe), cerebral amyloid angiopathy (none, mild, moderate, and severe), infarcts and lacunes (no and yes), and microinfarcts (no and yes).

**Result:**

Following exclusions, the final analytic sample included 2558 participants, including brains with none (n = 370), mild (n = 909), moderate (n = 884), and severe (n = 395) B‐ASC severity. The mean age at death was 82.3 years and half were females. More than half of the participants had severe ADNC (Braak NFT stages V or VI). Over 28% of included participants had TDP‐43 pathology in hippocampus, whereas 16.4% had HS‐A. The associations between B‐ASC and both LATE‐NC and HS‐A were strong and specific when controlling for other factors such as age (P<0.001). This association remained robust when a subset analysis was performed only analyzing results from individuals who died after 2014.

**Conclusion:**

There is a strong, replicable association between B‐ASC and LATE‐NC/HS‐A in the NACC Neuropathology Data Set. Microvasculopathy appears to be a component of the LATE‐NC/HS‐A brain condition, which is remarkable because both B‐ASC and LATE‐NC are strongly associated with “Alzheimer’s‐type” amnestic dementia.

